# Over-the-counter medications containing diphenhydramine and doxylamine used by older adults to improve sleep

**DOI:** 10.1007/s11096-017-0467-x

**Published:** 2017-05-02

**Authors:** Olufunmilola Abraham, Loren Schleiden, Steven M. Albert

**Affiliations:** 1 Department of Pharmacy and Therapeutics, University of Pittsburgh School of Pharmacy, 693 Salk Hall, 3501 Terrace St., Pittsburgh, PA 15261 USA; 2 Department of Behavioral and Community Health Sciences, University of Pittsburgh Graduate School of Public Health, 130 De Soto St., Pittsburgh, PA 15261 USA

**Keywords:** Beers Criteria, Over-the-counter medications, OTCs, Older adults, Sleep aids, Sleep problems, USA

## Abstract

*Background* The unintentional misuse of over-the-counter sleep aids among older adults is an important public health problem and a focus of Healthy People 2020. Accordingly, the 2015 Beers Criteria for Potentially Inappropriate Medication Use in Older Adults recommends that individuals 65 years or older avoid use of diphenhydramine and doxylamine; however, many over-the-counter sleep products contain these active ingredients. *Objective* To identify the proportion of older adults using an over-the-counter medication containing diphenhydramine or doxylamine, and compare their characteristics with older adults using an over-the-counter medication that does not contain these ingredients. *Setting* Study participants were recruited from the Community Registry of the Pittsburgh Claude D. Pepper Older Americans Independence Center. *Method* The study sample was taken from a larger survey of 1025 participants on sleep health and over-the-counter sleep medication use conducted from February to April 2015. A subset of 169 participants aged 65 and older reporting taking at least one over-the-counter product to improve sleep within the past 30 days (16.5%) were selected for our analysis on associations between participant characteristics and potentially inappropriate use of over-the-counter sleep medications. *Main outcome measure* The proportion and characteristics of older adults taking at least one over-the-counter medication containing diphenhydramine or doxylamine. *Results* Of the 223 over-the-counter sleep medications listed by participants, 115 (52%) contained diphenhydramine or doxylamine. Using the Beers Criteria, we found that more than half of participants (59%) had used a potentially inappropriate over-the-counter medication containing diphenhydramine or doxylamine to improve sleep within the past 30 days. Participants taking at least one diphenhydramine or doxylamine containing medication were less likely to be aware of any safety risks in taking over-the-counter sleep medications than participants not taking these products (38 vs 49%, *p* = 0.016). *Conclusion* A majority of older adults in a limited sample from the United States taking an over-the-counter medication to improve sleep are taking a product containing diphenhydramine or doxylamine, both of which are classified as potentially inappropriate for older adults. Awareness of the safety risks of over-the-counter medications and addressing conditions that impact sleep quality could be facilitated through consultation with pharmacists and other healthcare providers.

**Electronic supplementary material:**

The online version of this article (doi:10.1007/s11096-017-0467-x) contains supplementary material, which is available to authorized users.

## Impacts on practice

- A large number of adults in the United States experience sleep difficulties and primarily use OTC medications to manage this problem.
- Older adults, in particular, may be unaware of the safety risks of OTC sleep medications containing diphenhydramine and doxylamine.
- Consultation with appropriate and accessible health professionals, such as community pharmacists during time of purchase, could help to curtail potentially inappropriate use of OTC sleep medications.

## Introduction

Insufficient and poor quality of sleep is a prevalent health concern for many adults [1–3], and particularly in the elderly because of the increased risk of adverse health effects including hypertension, diabetes, obesity, stroke, and heart attack [4]. The National Sleep Foundation recommends a healthy sleep duration of 7–9 h for adults aged 26–64 years, and 7–8 h of sleep for adults aged 65 years and older [5]. A recent study found that more than one third of adults have reported a sleep duration of <7 h, which increases the risk for several detrimental health conditions and impaired cognitive functioning [4, 6]. More specifically, 13.7–37.75% of older adults in international studies reported they had experienced sleep problems [7–10]. In order to improve sleep and treat sleep problems, many older adults use over-the-counter (OTC) medications [11, 12].

Older adults in particular are major consumers of OTC medications for a variety of indications, and they account for 35% of OTC medication use in the United States [13]. A previous study found that the typical older adult in the United States uses as nearly as many OTC medications as prescription medications, which greatly increases the potential for harmful drug–drug interactions [14]. An increasing number of older adults opt for self-management of sleep difficulties, and a study reported that 12% of older adults (≥65 years old) in the United States use OTC sleep medications [15]. Almost half (44%) of older adults in the United States experience disturbed sleep at least a few nights each week and frequently use OTC sleep medications [16], however this self-care approach may not address the physiological changes, reductions in health status, loss of physical function, primary sleep disorders, psychological influences, or other underlying health conditions which may be a cause of sleep disturbances [17].

The unintentional misuse of OTC medications for improvement of sleep has been shown to be an important public health problem internationally and in the United States [18–21], and it is a focus of Healthy People 2020 [4, 22]. Concerns with medication safety have risen due to older adults’ use of OTC medications containing diphenhydramine or doxylamine (DIPH/DOX), which the Beers Criteria for Potentially Inappropriate Medication Use in Older Adults has deemed to be potentially inappropriate and unsafe for use in the elderly [23]. The Beers Criteria, published by the American Geriatrics Society, has been widely utilized and adapted in studies of potentially inappropriate medication use by older adults in other countries including Korea, Japan, Italy, and Spain [24–27]. A study indicated that older adults were twice as likely as younger adults to use OTC sleep medications containing antihistamines such as diphenhydramine or doxylamine for over 20 days [15].

OTC sleep medications containing DIPH/DOX increase the risk of hepatic and renal insufficiency, drug interactions, adverse events, and unintended anticholinergic effects in the elderly [4, 28]. OTC sleep medications containing DIPH/DOX are known to have anticholinergic properties that can result in cognitive impairments, hangover effects, dizziness, or falls, especially in older adults [16]. Furthermore, many older adults are concurrently taking prescription medications with anticholinergic properties such as tricyclic antidepressants. The combined anticholinergic burden of taking prescribed medications along with OTC medications containing DIPH/DOX increases the risk of adverse events and other unintended negative consequences [22]. Supplements that can be purchased as OTC sleep aids in the United States such as melatonin have also been increasingly used more to manage sleep problems [29]. There is a lack of evidence of the safety of melatonin for use on a chronic basis, and it is also considered a prescription drug in some countries [29]. OTC sleep medications are not intended for long-term use in managing complicated sleep problems such as insomnia, but rather for short-term treatment of sleep disturbance or deprivation.

Older adults rarely discuss sleep complaints with their healthcare providers or seek counseling from pharmacists when selecting or using OTC medications for sleep problems [30]. Healthcare providers and pharmacists are often unaware of OTC medication use by their patients, which may lead to duplication of therapies, potentially dangerous overdosing, and possible drug–drug or drug-disease interactions. Australian patients with insomnia regarded OTC sleep aids as safer than prescription sleep aids and felt that unclear labelling and instructions were leading to suboptimal use [31]. These misperceptions may be resolved through discussion with healthcare providers. Pharmacist-provided direct patient care has demonstrated favorable therapeutic, safety, and humanistic outcomes across various healthcare settings [32], and community pharmacists are accessible medication experts who may be able to consult patients about their sleep problems, potential non-pharmacological interventions, and safe OTC sleep aid use. Despite the widespread self-treatment with OTC sleep medications containing DIPH/DOX, the decision-making and selection process for using these medications has received very limited research attention.

Ensuring that older adults safely and effectively use or avoid using OTC sleep medications is critical in order to minimize potential drug–drug interactions and unintentional misuse. Nevertheless, we know surprisingly little about the characteristics of older adults who use potentially inappropriate medications to address sleep disturbances, their knowledge of OTC sleep aids, and the safety risks of the types of products being used.

## Aim of the study

The purpose of this study was to explore OTC medication use by older adults to improve sleep, by identifying the proportion and characteristics of participants using at least one OTC medication containing DIPH/DOX with participants using an OTC medication that does not contain DIPH/DOX.

## Ethics approval

Ethical approval for this study was granted by the University of Pittsburgh Institutional Review Board.

## Methods

### Study sample

The sample for this study was identified from participants of a previous mail survey on sleep health and use of OTC sleep medications among older adults from the Pittsburgh Claude D. Pepper Older Americans Independence Center. The Pepper Community Registry includes over 2000 community-dwelling persons from the Pittsburgh region aged 60 years and older who have consented to be contacted for participation in Pepper-approved research studies. The survey was originally sent via mail to 2064 Pepper registrants in February 2015. A total of 1025 participants returned completed surveys, and a subset of 169 participants aged 65 and older who reported taking at least one OTC sleep medication within the past 30 days (16.5%) were identified and selected for this study and further analyses. Informed consent was obtained from study participants.

### Measures

The mail survey was developed to collect information and characteristics of sleep quality in older adults, along with healthcare seeking behaviors including use of treatments or products used to alleviate sleep problems (primarily OTC sleep medications). To elicit responses indicating the use of OTC medications to improve sleep, participants were asked: “Do you use any over the counter (OTC) medications to help you fall asleep or stay asleep (OTC drugs include Benadryl, Tylenol PM, etc.)?” Participants were also asked about any prescription medications taken to help fall or stay asleep. The names, frequency of use, knowledge of safety risks and active ingredients, and satisfaction with use were collected for all OTC and prescription sleep medications reported. Survey items relating to consultation with a healthcare professional about choosing an OTC product for sleep and overall satisfaction with sleep (very dissatisfied, dissatisfied, neither satisfied nor dissatisfied, satisfied, very satisfied) were also collected. Survey responses were linked to participant demographics and basic health information collected by the Pepper registry (see “Supplementary Material”). This enabled the comparison of survey responses to participant demographic characteristics of interest including age, sex, marital status, race/ethnicity, and education.

### Analyses

We hypothesized that at least 50% of older adults used a potentially inappropriate OTC medication containing DIPH/DOX according to the Beers Criteria, and would be unaware of OTC medication safety risks [23]. OTC medications were evaluated for product name, indication(s), and active ingredient(s), and were categorized according to the following therapeutic characteristics and indications: sleep aid, combination sleep aid and pain reliever/fever reducer, pain reliever/fever reducer, allergy reliever, herbal, pain reliever/antacid, anticonvulsant/neuropathic pain reliever/anxiety reliever, vitamins, and dry mouth reliever. Analyses sought to identify associations between the use of OTC sleep aids containing DIPH/DOX and demographics, consultation with healthcare professionals, characteristics of OTC sleep aid use, health literacy, and the use of prescription medications to improve sleep (α = 0.05). All statistical analyses were performed using STATA software version 13.0 (StataCorp, College Station, TX).

## Results

### Baseline characteristics

Table 1 shows the baseline characteristics of the sample of 169 participants. Majority of participants were non-Hispanic White (95%), female (65%), married (58%), and had more than a 4-year college degree (52%).

**Table 1:** Baseline characteristics of participants who had used OTC sleep aids in the past 30 days

	Did not list any OTC products containing diphenhydramine or doxylamine n = 70 (41.4%)	Listed at least one OTC product containing diphenhydramine or doxylamine n = 99 (58.6%)	*p* value
Age			*p* = 0.779
65–69	9 (12.9)	15 (15.2)	
70–74	16 (22.9)	24 (24.2)	
75–79	16 (22.9)	23 (23.2)	
80–84	9 (12.9)	15 (15.2)	
85–89	10 (14.3)	15 (15.2)	
90+	10 (14.3)	7 (7.1)	
Female	45 (64.3)	65 (65.7)	*p* = 0.854
Marital status			*p* = 0.475
Single	8 (11.4)	5 (5.2)	
Married	38 (54.3)	60 (61.9)	
Widowed	17 (24.3)	22 (22.7)	
Divorced/separated	7 (10.0)	10 (10.3)	
Race/ethnicity			*p* = 0.348
Non-Hispanic white	69 (98.6)	92 (92.9)	
Non-Hispanic black	1 (1.4)	5 (5.1)	
Non-Hispanic other	0 (0.0)	2 (2.0)	
Education			*p* = 0.121
HS degree/GED or less	5 (7.3)	18 (18.4)	
Some college to 4-year degree	25 (36.2)	32 (32.7)	
More than 4 year college degree	39 (56.5)	48 (49.0)	
Missing/skipped	1 (0.1)	1 (0.1)	

### Characteristics of participants using OTC products containing DIPH/DOX

As shown in Table 2, more than half (59%) of the 169 survey participants who reported using an OTC sleep aid in the past 30 days had used at least one OTC medication containing DIPH/DOX, and 15 participants (9%) reported taking more than one OTC medication containing DIPH/DOX. Participants who reported taking at least one OTC medication containing DIPH/DOX were less likely than those taking other OTC sleep medications to report being aware of any safety risks (38 vs 49%, *p* = 0.016). There were no significant differences between DIPH/DOX users and participants taking only OTC medications not containing DIPH/DOX to improve sleep in terms of consulting a pharmacist or physician when choosing OTC sleep medication (28 vs 40%, *p* = 0.111), experience of side effects from using OTC sleep aids (15 vs 9%, *p* = 0.202), knowledge of the active ingredient in their most recently used OTC sleep aid (57 vs 43%, *p* = 0.066), satisfaction with their OTC sleep aids (41 vs 48%, *p* = 0.187), obtaining assistance with reading hospital or pharmacy materials (16 vs 19%, *p* = 0.673), difficulty learning about medical conditions (15 vs 20%, *p* = 0.427), or using a prescription medication to help fall or stay asleep (19 vs 26%, *p* = 0.329). Of the 56 participants who had consulted a pharmacist or physician when choosing their OTC sleep medication, the majority had consulted a physician (80%), with 21% consulting a pharmacist and about 5% reporting that they consulted a nurse practitioner.

**Table 2:** Characteristics of participants using OTC products containing DIPH/DOX

	Did not list any OTC products containing diphenhydramine or doxylamine n = 70 (41.4%)	Listed at least one OTC product containing diphenhydramine or doxylamine n = 99 (58.6%)	*p* value
Did you consult your pharmacist or doctor when choosing this OTC medication for sleep?			*p* = 0.111
Yes	28 (40.0)	28 (28.3)	
No	42 (60.0)	71 (71.7)	
If yes (*n* = 56), who did you consult? (not mutually exclusive)			
Doctor (yes vs no)	23 (82.1)	22 (78.6)	
Pharmacist (yes vs no)	5 (17.9)	7 (25.0)	
Nurse practitioner (yes vs no)	2 (7.1)	1 (3.6)	
Did you experience any side effects from using these over the counter (OTC) medications?			*p* = 0.202
Yes	6 (8.6)	15 (15.2)	
No	64 (91.4)	84 (84.9)	
Do you know the active ingredient(s) contained in your most recently used OTC medication for sleep? (*missing* = 7)			*p* = 0.066
Yes	30 (42.9)	55 (57.3)	
No	40 (57.1)	41 (42.7)	
Are you aware of any safety risks in taking OTC sleep medications? (*missing* = 4)			*p* = 0.016
Yes	34 (48.6)	38 (38.4)	
No	36 (51.4)	61 (61.6)	
How satisfied are you with using this/these OTC sleep aid(s) to improve your sleep quality? (*missing* = 3)			*p* = 0.187
Satisfied (very satisfied—neither nor)	41 (48.2)	52 (40.9)	
Not satisfied (dissatisfied, very dissatisfied)	44 (51.7)	75 (59.1)	
How often do you have someone (like a family member, friend, hospital/clinic worker, or caregiver) help you read hospital or pharmacy materials? (*missing* = 3)			*p* = 0.673
Occasionally/sometimes/often/always	13 (18.8)	16 (16.3)	
Never	56 (81.2)	82 (83.7)	
How often do you have problems learning about your medical condition because of difficulty understanding written information? (*missing* = 2)			*p* = 0.427
Occasionally/sometimes/often/always	14 (20.0)	15 (15.3)	
Never	56 (80.0)	83 (84.7)	
Do you ever use prescription medications to help you fall asleep or stay asleep?			*p* = 0.329
Yes	18 (25.7)	19 (19.4)	
No	52 (74.3)	79 (80.6)	
Did you experience any side effects from using these prescription medications? (*n* = 37) (*missing* = 4)			*p* = 0.601
Yes	3 (17.6)	1 (6.3)	
No	14 (82.4)	15 (93.8)	

As shown in Table 3, a similar number of men (58%) and women (59%) reported taking an OTC medication containing DIPH/DOX; however, a higher percentage of men (24%) than women (19%) were taking OTC medications containing DIPH/DOX more than 10 times per month. A lower proportion of participants over the age of 90 reported taking at least one medication containing DIPH/DOX (41%) compared to other age groups.

**Table 3:** OTC DIPH/DOX use by gender and age group

Overall (n = 169)	OTC DIPH/DOX use 99 (58.6)		OTC DIPH/DOX use for more than 10 days per month 35 (20.7)	
	Female (n = 110)	Male (n = 59)	Female (n = 110)	Male (n = 59)
Gender	65 (59.1)	34 (57.6)	21 (19.1)	14 (23.7)
Age group	Female	Male	Female	Male
65–69 (n = 24)	13 (65.0)	2 (50.0)	7 (35.0)	0 (0.0)
70–74 (n = 40)	17 (58.6)	7 (63.6)	3 (10.3)	3 (27.3)
75–79 (n = 39)	16 (59.3)	7 (58.3)	6 (22.2)	3 (25.0)
80–84 (n = 24)	6 (50.0)	9 (75.0)	2 (16.7)	2 (16.7)
85–89 (n = 25)	8 (57.1)	7 (63.6)	1 (7.1)	4 (36.4)
90 + (n = 17)	5 (62.5)	2 (22.2)	2 (25.0)	2 (22.2)

### OTC products used to improve sleep

Table 4 describes the 223 products listed by participants used to help fall or stay asleep, and the majority contained DIPH/DOX (52%). Very few OTC medications (8%) listed by participants as being used to help or fall asleep were determined to be solely classified as sleep aids; rather they were often combination products or classified in a different group, such as pain relievers/fever reducers or antihistamines. The most often-listed OTC medication by participants was Tylenol PM, whose active ingredients are diphenhydramine and acetaminophen. While nearly one third (29%) of OTC medications listed were combination sleep aid and pain reliever/fever reducers (for example, Tylenol PM, Advil PM), nearly as many (26%) reported products were indicated for pain relief/fever reduction, pain relief/antacid, or anticonvulsant/neuropathic pain relief/anxiety relief alone (such as, Tylenol, Aspirin). Products indicated for allergy relief (16%) typically contained diphenhydramine. Participants also listed herbal products (20%), 93% of which contained melatonin. Vitamins, dry mouth relievers, and “unable to be categorized” products together made up <2% of products listed.

**Table 4:** Group, active ingredients, and name of OTC products listed as helping to fall or stay asleep

Group	Active ingredient(s)	OTC product name	Frequency listed
Combination sleep aid and pain reliever/fever reducer	Diphenhydramine/acetaminophen	Tylenol PM	42
	Diphenhydramine/ibuprofen	Advil PM	10
		Ibuprofen PM	2
	Diphenhydramine/naproxen	Aleve PM	5
	Diphenhydramine/acetaminophen	Acetaminophen PM	2
		Aspirin PM	1
		Pain Reliever PM	1
	Doxylamine/acetaminophen/dextromethorphan	Nyquil	1
Herbal	Melatonin	Melatonin	38
		MidNite	2
		“Sleep Remedy”	1
	Humulus lupus, passiflora, chamomilla, avena sativa	Calms Forte Hylands	1
	Chamomile	Chamomile Tea	1
	Valerian	DeltaSom	1
	Melatonin/valerian	Somnapure	1
Antihistamine	Diphenhydramine	Benadryl	28
	Loratadine	Allergy relief	1
Sleep aid	Diphenhydramine or doxylamine	Diphenhydramine	6
		ZzzQuil	3
		Simply Sleep	2
		Nighttime	1
		“Sleep aid”	10
		Doxylamine Succinate	1
Pain reliever/fever reducer	Acetaminophen	Tylenol	23
		Acetaminophen	4
	Aspirin	Aspirin	8
	Ibuprofen	Ibuprofen	8
		Advil	4
	Naproxen	Aleve	7
		Naproxen	2
Pain reliever/antacid	Aspirin/sodium bicarbonate/anhydrous acid	Alka-Seltzer	1
Anticonvulsant/neuropathic pain reliever/anxiety reliever	Gabapentin	Gabapentin	1
Dry mouth reliever	Xylitol	Xylimelts	1
Not specified	Not Specified	Cough and Cold	1
		Onemovan Tea	1
		Vitamins	1

## Discussion

Results from this study suggest that many older adults are self-managing their sleep problems by taking OTC medications, many of which are not meant to be taken long-term by individuals of any age and may be inappropriate for older adults. Even among our highly educated sample, with the majority of participants having education beyond a 4-year college degree, 59% of respondents who had used an OTC product to improve sleep in the past 30 days were using a sleep medication containing DIPH/DOX. Furthermore, approximately one in five respondents were using a sleep medication containing DIPH/DOX more than 10 times in a month. These findings, along with previous research in the United States and France, suggest that many individuals are taking sleep aids more frequently or for a longer period of time than typically recommended by the healthcare professionals [33, 34].

The most commonly used OTC medications were combinations of diphenhydramine and a pain reliever or fever reducer such as Tylenol PM (acetaminophen and diphenhydramine), Advil PM (ibuprofen and diphenhydramine), and Aleve PM (naproxen and diphenhydramine). Products containing only DIPH/DOX as an active ingredient were also listed, such as Benadryl, which is meant to relieve allergy or common cold symptoms, and ZzzQuil, which is specifically targeted towards helping a person fall and stay asleep. Diphenhydramine has potentially harmful effects on older adults including risk of motor impairment [35] and anticholinergic effects [36, 37]; its use is also associated with falls [38]. Diphenhydramine may be especially dangerous in older adults due to its longer half-life compared younger individuals [39–41]. Another safety risk of using diphenhydramine at night is the presence of a residual sedative effect the morning after use [42].

In addition to OTC products containing DIPH/DOX, participants listed herbal products, most of which contained melatonin, as being used for improving sleep. The United States Food and Drug Administration (FDA) considers these products to be dietary supplements and therefore distinct from OTC sleep medications. Without FDA regulation, there are concerns with ingredients in melatonin sold over the counter with a recent study finding certain brands containing anywhere from 1% of melatonin claimed on the label to more 47%, with some brands containing higher amounts of arsenic than the recommended limit [43]. While melatonin has been shown to be well-tolerated in clinical trials in older patients and safe for short term use, there is little evidence to support its effectiveness in treating primary and secondary sleep disorders [44–46]. Participants also listed other OTC products taken to help fall or stay asleep, including many products that do not contain any hypnotic active ingredients and are not necessarily intended to be taken to improve sleep. The prevalent use of these products leads us to believe that pain or discomfort caused by other conditions is negatively impacting sleep in our sample of older adults. Persistent pain is another potential area for intervention by healthcare professionals, such as community pharmacists, who could provide counseling to discuss avoidance of combination sleep aid/pain relievers with older adult patients and refer them to resources for appropriate pain management.

Participants who used OTC products not containing DIPH/DOX were otherwise fairly similar on most measured factors to those who used DIPH/DOX products, with the exception that participants using DIPH/DOX products were less aware of any safety risks in taking OTC sleep medications. This association may suggest the possibility that older adults would decrease their use of certain inappropriate OTC sleep medications with increased awareness of the associated safety risks. Consultation with a healthcare professional such as a physician or community pharmacist could facilitate this awareness in older adult patients. Most of the participants in this study did not consult a healthcare professional when choosing an OTC medication for sleep problems, and if they did it was primarily a physician, not a pharmacist. Community pharmacists are easily accessible where most OTC sleep medications are sold and purchased without older adults having to make an appointment, and can engage these patients in discussions about their sleep issues, selection of OTC products, or avoidance of unsafe use [47]. A community pharmacist could suggest alternative sleep hygiene modifications or refer patients to a physician so that sleep problems could be addressed under medical supervision.

While a patient may choose an OTC product to treat their sleep problems, sleep disturbances may be caused by underlying conditions such as depression, anxiety, pain, or sleep apnea. Only treating sleep problems may leave these potentially serious conditions unresolved. Consultation by community pharmacists with older adult patients could elicit underlying conditions which manifested through sleep problems and then refer patients to their physicians, rather than the individual self-managing these symptoms. In Australia for instance, sedating antihistamines such as DIPH/DOX are classified as “Pharmacist Only Medicines,” and their purchase requires interaction with a pharmacist [48]. A study using simulated patients found that Australian pharmacists typically asked about problems related to sleep and provided adequate counseling when a product was supplied [48]. A similar model incorporating required interactions with pharmacists in order to obtain certain OTC sleep aids may be beneficial if implemented in the United States. In a recent study of medication therapy management (MTM) intervention, community pharmacists were found to be effective in reducing the use of fall-risk inducing medications (FRIDs) among older adults, as over 75% of older adults receiving the MTM intervention stopped using all FRIDs [49]. These study findings indicate that community pharmacists can have the potential to positively influence safe use of medications by older adults in other areas, particularly potentially inappropriate OTC sleep medication use.

The prevalence of unhealthy sleep duration in one third of adults [6] may be contributing to the rise in use of OTC sleep medications. The pervasiveness of sleep disturbance indicates that it is important for healthcare professionals, particularly community pharmacists, to consistently assess patients for sleep concerns, educate older adults about behavior changes to improve sleep hygiene, and emphasize safe and effective use of OTC sleep medications. Since older adults often do not discuss sleep problems with healthcare professionals, discussions around sleep quality and management of sleep problems should be included in regular health assessments and patient care in clinical practice.

### Limitations

This study was limited by its cross-sectional design because we collected data at one point in time. Our sample, including registry members who were primarily white and had a high level of education, is not representative of all older adults. This study was also conducted in one urban region of Western Pennsylvania in the United States, thus results may not be generalizable to other populations in different countries. Selection bias may have impacted our results, as only those who voluntarily filled out and returned the survey via mail were able to be included in the study. We relied on participants to remember and accurately report OTC medication use which could vary due to possibility of recall bias or inaccuracy of responses to survey items. Moreover, our list of elicited OTC sleep medications contains many products not designed to facilitate sleep. Other methods, such as semi-structured interviews, could further clarify which products were being taken and for what specific purposes.

## Conclusion

Study findings indicate the proportion of OTC medications used by older adults to improve sleep and potential for unsafe use. A majority of participants reported taking an OTC sleep medication containing DIPH/DOX, which the Beers Criteria has classified as potentially inappropriate for use among older adults. OTC medications are also being taken to relieve the symptoms of other conditions such as pain that would contribute to trouble falling or staying asleep. Many older adults are unaware of the safety risks of OTC products containing DIPH/DOX, which sheds light on the importance of community pharmacists and other healthcare professionals educating them on appropriate sleep hygiene and safe use of medications.

## Electronic supplementary material

Below is the link to the electronic supplementary material.

Supplementary material 1 (DOCX 26 kb)
