# Supplementary material for: Over-the-counter medications containing diphenhydramine and doxylamine used by older adults to improve sleep
Source: Int J Clin Pharm. 2017 May 2;39(4):808–17. doi: 10.1007/s11096-017-0467-x (PMC5541127; doi:10.1007/s11096-017-0467-x)
Supplement: Supplementary file 1 — Supplementary material 1 (DOCX 26 kb) [file 11096_2017_467_MOESM1_ESM.docx]

**Appendix**

*Survey Questions*

Q1a Do you use any **over the counter (OTC)** medications to help you **fall asleep** or **stay asleep**? *(OTC drugs include Benadryl, Tylenol PM, etc...)*

- Yes
- No

Q1b **If Yes**, provide the following for each OTC medication you have used to help you fall asleep or stay asleep:

|  | OTC Medication | | Number of Times used per month (select one) | | | Choose any that apply | |
| --- | --- | --- | --- | --- | --- | --- | --- |
|  | Name of Substance/Product | Describe how you use it (dosing/frequency) | <5 | 5-10 | >10 | Used to fall asleep | Used to stay asleep |
| 1 |  |  |  |  |  |  |  |
| 2 |  |  |  |  |  |  |  |
| 3 |  |  |  |  |  |  |  |
| 4 |  |  |  |  |  |  |  |
| 5 |  |  |  |  |  |  |  |

Q2 Have you used one of the above listed **over the counter (OTC)** medications to help you fall asleep or stay **asleep in the last 30 days**? *(OTC drugs include Benadryl, Tylenol PM, etc...)*

- Yes
- No

Q3a Do you know the active ingredient/s contained in your most recently used **OTC medication for sleep**?

- Yes
- No

Q3b **If Yes**, please list here:

|  |
| --- |

Q4a Did you consult your **pharmacist** or **doctor** when choosing this **OTC medication for sleep**?

- Yes
- No

Q4b **If Yes**, who did you consult? (Check all that apply)

- Doctor
- Pharmacist
- Nurse Practitioner
- Family member
- Other (please specify) ____________________

Q5a Do you believe there are any **safety** **risks** in taking **OTC** **sleep** **medications** (e.g., drug interactions with prescribed medications) whether or not you use these medications?

- Yes
- No

Q5b **If yes**, please describe any safety risks you are aware of below:

|  |
| --- |

Q6 How satisfied are you with using this/these **OTC** **sleep** **aid(s)** to improve your sleep quality?

- Very Dissatisfied
- Dissatisfied
- Neither Satisfied nor Dissatisfied
- Satisfied
- Very Satisfied

Q7 Do you **ever** use **prescription** medications to help you **fall** **asleep** or **stay** **asleep***? (Prescription medications include Ambien, Valium, etc...)*

- Yes
- No

Q8 **If Yes**, provide the following for each prescription medication you have used to help you fall asleep or stay asleep:

|  | Prescription Medication | | Number of Times used per month (select one) | | | Choose any that apply | |
| --- | --- | --- | --- | --- | --- | --- | --- |
|  | Name of Substance/Product | Describe how you use it (dosing/frequency) | <5 | 5-10 | >10 | Used to fall asleep | Used to stay asleep |
| 1 |  |  |  |  |  |  |  |
| 2 |  |  |  |  |  |  |  |
| 3 |  |  |  |  |  |  |  |
| 4 |  |  |  |  |  |  |  |
| 5 |  |  |  |  |  |  |  |

Q9 Have you used one of the above listed prescription medications to help you fall asleep or stay asleep in the last 30 days?

- Yes
- No

Q10a Did you experience any side effects from using these prescription medications?

- Yes
- No

Q10b **If Yes**, please describe these side effects (Day-time drowsiness, fragmented sleep, etc…):

|  |
| --- |

Q11a Do you ever use any other prescription medications?

- Yes
- No

Q11b **If Yes**, please list the **prescription** **medications** you are currently taking, what you are taking them for, and describe how you use these medications:

|  | Name of Substance/Product | What you are taking it for? | How much do you take? | How often do you take it? |
| --- | --- | --- | --- | --- |
| ***e.g.,*** | ***Lipitor (atorvastatin)*** | ***high cholesterol*** | ***1 tablet*** | ***Once daily*** |
| 1 |  |  |  |  |
| 2 |  |  |  |  |
| 3 |  |  |  |  |
| 4 |  |  |  |  |
| 5 |  |  |  |  |
| 6 |  |  |  |  |
| 7 |  |  |  |  |
| 8 |  |  |  |  |

Q12 How often do you have someone (like a family member, friend, hospital/clinic worker, or caregiver) **help you read hospital or pharmacy materials**?

🔾 Always

🔾 Often

🔾 Sometimes

🔾 Occasionally

🔾 Never

Q13 How often do you have problems learning about your medical condition **because of difficulty understanding written information**?

🔾 Always

🔾 Often

🔾 Sometimes

🔾 Occasionally

🔾 Never

*Participant Demographics*

**Birth Date:** ______ ______ ______ **Age:** ______ **Gender:** □ Male □ Female

Month Day Year

**Ethnic Category:** □ Hispanic □ Not Hispanic or Latino

**Racial Categories:** □ American Indian/Alaska Native

□ Asian

□ Native Hawaiian or Other Pacific Islander

□ Black or African American

□ White

□ other (specify) ___________

**Marital Status:** □ Single □ Separated □ Divorced

□ Married □ Widowed □ Living with partner

**What is the highest grade or year of school you have ever completed, including trade school or vocational school, or college?** □ 0-12 □ no schooling □ GED

College: Vocational School: □ Graduate or Professional

□ 1 year □ 1 year

□ 2 years □ 2 years □ Refuse to answer

□ 3 years □ 3 years

□ 4 years
